# Beneficial Effects of Water-Based Exercise Alone and in Combination with Cognitive Training on Cardiovascular Fitness and Arterial Stiffness in Older Adults with Mild Cognitive Impairment: A Randomized Controlled Trial

**DOI:** 10.3390/life15081195

**Published:** 2025-07-28

**Authors:** Patcharee Kooncumchoo, Sutaya Meekum, Somrudee Harnmanop, Nongnuch Luangpon, Kornanong Yuenyongchaiwat

**Affiliations:** 1 Department of Physical Therapy, Faculty of Allied Health Sciences, Thammasat University, 99 Moo 18, Paholyothin Road, Klong Luang, Pathumtani 12120, Thailand; 2 Center of Excellence in Creative Engineering Design and Development, Thammasat University, 99 Moo 18, Paholyothin Road, Klong Luang, Pathumtani 12120, Thailand; 3 Department of Physical Therapy, Faculty of Allied Health Sciences, Burapha University, 169 Long Had Bangsaen Rd, Saen Suk, Chonburi 20131, Thailand; 4 Thammasat University Research Unit for Physical Therapy in Respiratory and Cardiovascular Systems, Thammasat University, 99 Moo 18, Paholyothin Road, Klong Luang, Pathumtani 12120, Thailand

**Keywords:** mild cognitive impairment, cardiovascular fitness, arterial stiffness, water exercise, cognitive training, older adults

## Abstract

Mild cognitive impairment (MCI) is the transitional stage between normal cognition and dementia and is associated with arterial stiffness, which may lead to cardiovascular disease. A water-based exercise (W) presents a low-impact activity for the joints and increases resistance compared to exercises performed in the air, which benefits older adults. However, little evidence has been found regarding the effect of W on promoting cognitive and physical performance in older individuals with MCI. Therefore, this study aimed to investigate and compare the post-training effects of W alone and in combination with cognitive training on cognitive function, cardiovascular fitness, and arterial stiffness in older adults with MCI. Forty-six adults with MCI, aged 65 years or older, were enrolled. Participants were divided into two groups: a W group and a water-based exercise combined with cognitive training (W-COG) group. Both groups performed an aerobic exercise program in water for 60 min per/day, 3 day/week, for 12 weeks. Participants in the W-COG group simultaneously performed aerobic exercise and cognitive training in water. Cognitive performance, cardiovascular fitness, and arterial stiffness were examined before and after the intervention. The results revealed improvements in cognitive performance and cardiovascular fitness in both the W and W-COG groups after 12 weeks of intervention. However, there were no significant differences in cognitive and cardiovascular fitness changes between the two groups. Neither the W nor the W-COG groups showed a decrease in brachial pulse wave velocity. Therefore, W interventions have the potential to enhance cognitive function, restore cognition, and improve cardiovascular fitness in older adults with MCI.

## 1. Introduction

Older adults are often associated with the deterioration of the musculoskeletal and cardiopulmonary systems, and neurodegenerative diseases, including dementia [1,2]. Mild cognitive impairment is defined as the “symptomatic pre-dementia stage” on the continuum of cognitive decline in older adults and can reflect the early stage of dementia [3]. The rate of progression from mild cognitive impairment to dementia is 10.0% per year; additionally, an annual rate of 3% per year has been reported in community-dwelling older adults with mild cognitive impairment [4].

Prior studies have shown that arterial stiffness is associated with an increased risk of cardiovascular disease [5,6,7]. Furthermore, an association between cognitive performance and arterial stiffness has been reported in older adults with higher arterial stiffness and lower cognitive function [8,9,10,11,12]. A systematic review and meta-analysis of 29 observational and nine longitudinal studies showed a negative relationship between pulse wave velocity and cognitive performance after controlling for age, sex, educational level, depression scale score, and cardiovascular risk factors [13]. Additionally, executive function, memory, and global cognition were negatively associated with arterial stiffness [13]. Furthermore, cognitive impairment is associated with increased arterial stiffness and an increased risk of cardiovascular disease [14,15,16]. Systematic atherosclerosis was shown to contribute to cognitive function and was directly associated with the progression of Alzheimer’s disease.

There are many treatments, both pharmacological and non-pharmacological, available for delaying the progression to dementia in older adults with mild cognitive impairment. Exercise is an alternative non-pharmacological treatment. Previous research has shown that moderate aerobic exercise has a positive effect on cognitive performance [17,18,19]. Additionally, physical exercise can improve cognitive function in healthy older individuals with cognitive impairment [20,21]. In a randomized controlled trial, Erickson et al. [22] examined older adults who underwent aerobic exercise training for 6 months. Aerobic exercise training increased the volume of the left and right anterior hippocampus, resulting in measurable improvements in spatial memory performance. In addition, exercise increased levels of brain-derived neurotrophic factor in the hippocampus, and this volumetric increase was positively associated with elevated serum levels of brain-derived neurotrophic factor, a key regulator of neurogenesis within the dentate gyrus. In contrast, individuals in the control group exhibited a decline in hippocampal volume over time [22]. Therefore, exercise can reduce arterial stiffness and improve cognitive performance in older adults [22,23,24].

Previous studies have suggested that combined physical and cognitive exercise (dual-task) can improve cognitive abilities, logical memory, and physical performance in elderly individuals with mild cognitive impairment [25]. Furthermore, dual-task memory and aerobic exercises may improve balance and gait in older adults with mild cognitive impairment [26]. A systematic review suggested that combined cognitive and physical interventions may be more effective in promoting functional activities among older adults with mild cognitive impairment than cognitive training or single physical training alone [27].

Water-based exercise is an alternative program for enhancing the health of elderly individuals. The buoyancy of water decreases the compressive forces within the joints while offering hydrostatic support to the upright position [28,29]. Furthermore, exercising in water promotes greater and more intense movements in the older adult population. The specific properties of aquatic exercises are buoyancy and water viscosity, which promote greater assistance or resistance during movement and encourage participants to move over a wider range while exercising in water [30].

Furthermore, a beneficial effect of water-based exercises on cognitive function has been observed in healthy individuals [31]. There is evidence supporting the beneficial effects of water-based exercise in promoting cognitive function, cardiovascular fitness, and arterial stiffness in older adults with mild cognitive impairment. Therefore, this study aimed to explore the benefits of water-based exercises and water-based exercises combined with cognitive training on cardiovascular fitness and arterial stiffness in older adults with mild cognitive impairment.

## 2. Materials and Methods

### 2.1. Participants and Design

The sample size was calculated using the G-power version 3.1.9.4 program, with the confidence intervals and power set at 95% and 80%, respectively. The significance level for changes was accepted at 0.05. The required study population was determined to be 40 individuals, and an additional 15% were included to account for potential dropouts. Therefore, the total number of participants in this study was 46, with 23 participants assigned to each group.

Ambulatory older adults in the community were screened for mild cognitive impairment using the Montreal Cognitive Assessment (MoCA), and their independence in activities of daily life was assessed using the Lawton Instrumental Activities of Daily Living Scale [32]. Participants in the community with mild cognitive impairment aged 65 years (both males and females) were enrolled. Individuals with MoCA scores ranging between 17 and 24 points (depending on education level: a full score was 30 points) were considered to have mild cognitive impairment. Participants who had been diagnosed with mental illness, uncontrollable cardiorespiratory disease (e.g., exacerbation of chronic obstructive pulmonary disease or heart disease), or musculoskeletal pain were excluded. Participants with contraindications for water-based exercises, such as open wounds, urinary incontinence, and hydrophobia, were also excluded.

### 2.2. Procedures

This was a single-blind randomized controlled trial. Participants were randomly assigned to one of two groups: a water-based exercise group and a water-based exercise combined with cognitive training group. A moderate-intensity exercise (50–70% of maximum heart rate, which was defined as 220 minus the age) protocol was applied in both groups for 60 min per day, three days per week, over a 12-week period. During the exercise program, a smartwatch was attached to the participant to monitor their heart rate.

The protocol for the water-based exercises group consisted of 10 min of warm-up (e.g., stretching of the upper limb and trunk muscles, stretching of the lower limb muscles, and breathing exercises with arm movement upward/sideways). In addition, a 40-min aerobic exercise that consisted of hip flexion and abduction, side back kick, alternate high knee, squat and rise up on toe, back extension with alternate hip extension, back extension with hip abduction and external rotation, raising arm in diagonal direction, squat punch and kick, three-side-step and reach with dumbbell, step jack sideways and forward with a dumbbell, squat with lower arm down in diagonal direction, and squat with horizontal shoulder abduction was performed. The exercise concluded with a 10-min cool-down (same protocol as the warm-up).

The water-based exercise combined with cognitive training group started with a 10-min warm-up (same as the water-based exercise group) and then performed a 40-min aerobic exercise, simultaneously with cognitive training involving recalling a number and color, and a 10-min cool-down. The details of the aerobic exercise were the same as those for the water-based exercise group.

The cognitive performance, cardiovascular fitness, and arterial stiffness of the participants before and after the 12-week program was evaluated by assessors with 2 years of physical therapy experience. Cardiorespiratory fitness was measured using the 2-min step test, a tool for assessing functional fitness introduced by Rikli and Jones [33]. The participants were instructed to stand next to a wall; marks were indicated at the height of the iliac crest and patella, and half of the distance between the two sites was also marked. All participants were asked to raise each knee to the mark on the wall as many times as possible for 2 min. Scores were calculated when the right knee reached the required height [33].

Brachial pulse wave velocity was used to assess arterial stiffness. The brachial pulse wave velocity was measured using the VP-1000 Plus (Omron Healthcare, Osaka, Japan). While carotid–femoral pulse wave velocity is the gold standard for assessing arterial stiffness, brachial pulse wave velocity correlates with carotid–femoral pulse wave, with a correlation of r = 0.755 [34]. The participants adopted a supine position and rested quietly for 10 min. Following this, pressure cuffs were placed on both arms (i.e., right and left brachial cuff) and ankles (left and right cuff) to measure the pulse waves of the brachial and posterior tibial arteries, respectively.

### 2.3. Data Analysis

Statistical analyses were performed using IBM SPSS Statistics for Windows version 24. Descriptive statistics were used to analyze the general participant characteristics. Data are presented as the mean and standard deviation (SD) or standard error of the mean (SEM). For the intention-to-treat analysis, a two-way mixed repeated analysis of variance was used to analyze the variables, both within and between the groups. A post hoc analysis was performed using the Bonferroni test with the statistical significance level set at 0.05.

## 3. Results

**Figure 1:**
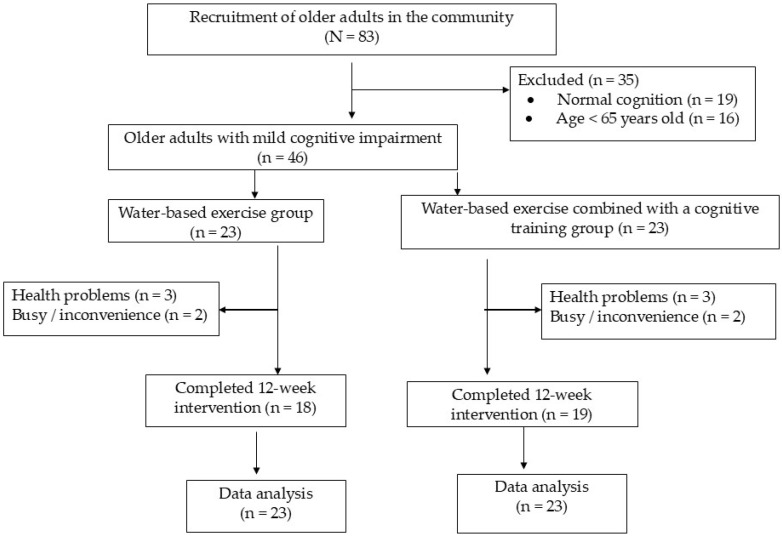
The diagram shows the flow of participants in the study.

Table 1 shows the demographic characteristics of the participants in the water-based exercise and water-based exercise combined with cognitive training groups. Forty-six elderly individuals with mild cognitive impairment were divided into two groups using a simple random sampling technique: water-based exercise (*n* = 23) and water-based exercises combined with cognitive training (*n* = 23). After the 12-week intervention period, five participants dropped out of the water-based exercise group and four dropped out of the water-based exercise combined with cognitive training group because of health problems or inconvenience in continuing the program (Figure 1). The adherence rates of participants in the water-based exercise and water-based exercise combined with cognitive training groups who completed 12 weeks of intervention were 78.26% and 82.61%, respectively.

The participant ages in the water-based exercise and water-based exercise combined with cognitive training groups were 68.3 ± 3.3 and 69.6 ± 3.8 years, respectively. Most participants had a primary education background: 72.2% in the water-based exercise group and 52.6% in the water-based exercise combined with cognitive training group. The number of underlying health conditions and regular exercise behaviors did not differ in both groups (*p* < 0.05). In addition, there were no significant differences in participant characteristics between the two groups (i.e., age, sex, body mass index, and educational attainment).

After 12 weeks of the intervention program, the MoCA scores increased to 3.78 and 4.09 in the water-based exercise group and water-based exercise combined with cognitive training groups, respectively, indicating an improvement in cognition in both groups. Fourteen participants (77.77%) in the water-based exercise group and seventeen (89.47%) in the water-based exercise combined with cognitive training reverted from mild cognitive impairment to normal cognition (defined as a MoCA score ≥ 25 points).

The 2-min step test scores significantly increased to 22.87 and 18.39 in the water-based exercise and water-based exercise combined with cognitive training groups, respectively. However, there was no significant improvement in cardiovascular fitness between the two groups. In addition, neither the water-based exercise nor the water-based exercise combined with cognitive training group showed significant differences in the brachial pulse wave velocity, within (F (1,44) = 0.061, *p* = 0.806 and F (1,44) = 2.163, *p* = 0.149, respectively) or between groups (F (1,44) = 2.896, *p* = 0.096, and F (1,44) = 3.300, *p* = 0.076, respectively) (Table 2).

## 4. Discussion

The benefits of water-based exercise and water-based exercise combined with cognitive training on cardiovascular fitness (measured by the 2-min step test) and arterial stiffness (measured by brachial pulse wave velocity) were explored in older adults with mild cognitive impairment. The results showed that older adults with mild cognitive impairment who performed either water-based exercises or water-based exercises combined with cognitive training displayed cardiovascular fitness improvements after a 12-week intervention program. However, there were no improvements in cardiovascular fitness between the two groups. Additionally, there were no significant differences in decreased brachial pulse wave velocity (i.e., arterial stiffness) between or within groups.

Improvements in cardiovascular fitness and cognition in both groups were associated with moderate-intensity aerobic exercise. This study found an increase in cardiovascular fitness (measured by the 2-min step test) and cognitive performance in both water-based exercise and water-based exercise combined with cognitive training groups. Improvements in cardiovascular fitness and cognitive function have been previously demonstrated with moderate-intensity aquatic exercises in older adults [35,36,37,38]. In this study, the intensity was set at a moderate level, ranging from 50% to 70% of the maximum heart rate. Increased heart rate during moderate-intensity aerobic exercise leads to increased blood circulation, oxygen levels, and neurotransmitter and nutrient supply to the brain [39]. Additionally, moderate-intensity exercises can be adapted to neural and vascular functions, which leads to increased perfusion to exercising muscles due to changes in endothelial function and arterial compliance [40]. Neurophysiological changes are enhanced by neurogenesis, angiogenesis, synaptic plasticity, decreased proinflammatory processes, and reduced cellular damage due to oxidative stress [41]. Additionally, exercise, including water-based exercise, has been shown to increase neurotrophic factors such as brain-derived neurotrophic factor, which is related to increased cognitive performance in older adults [42,43,44]. This promotes cognitive function and cardiovascular fitness.

However, it should be noted that there was no observed effect of water-based aerobic exercise on arterial stiffness in this study. Okamoto and Hashimoto reported that isometric handgrip training for 5 days per week over 8 weeks can decrease pulse wave velocity and improve cognitive function in older adults [23]. However, moderate-to-vigorous aerobic exercise training over a 12-month program was found to reduce arterial stiffness and increase cerebral blood flow in patients with amnestic mild cognitive impairment [23] and in older adults [45]. Older adults who performed water-based aerobic exercise for 10 weeks, at 3 days/week for 60 min/day, with exercise intensity set at 50–75% of their heart rate reserve, showed decreased pulse wave velocity [46]. Moreover, a systematic review and meta-analysis of 11 randomized controlled trials indicated an effect of aerobic exercise on reducing pulse wave velocity in older adults.

A systematic review and meta-analysis of 18 studies including 775 older adults found that aerobic exercise >8 weeks, but not 4–8 weeks, improved pulse wave velocity in healthy older adults, but not in older adults with disease [47]. Ha et al. [48] reported a reduction in total cholesterol, triglycerides, high-density lipoprotein cholesterol, and low-density lipoprotein cholesterol levels in older women who performed aquarobic exercise or aquarobic exercise combined with burdock intake. However, no statistically significant differences in pulse wave velocity within or between groups were observed after completing aquarobic exercise, aquarobic exercise combined with burdock intake, or burdock intake after a 12-week intervention [48]. Notably, vigorous-intensity exercise has been shown to effectively reduce pulse wave velocity, but moderate-intensity exercise has not [49]. Therefore, it is plausible that the intensity of the water-based exercise program, which is a moderate-intensity exercise (50–70% of maximum heart rate) with a short duration, was insufficient to reduce pulse wave velocity. Further studies are warranted to explore the effects of different exercise modalities and intensities on arterial stiffness.

Another reason might be that the average pulse wave velocity in older adults aged >60 years is 16.3 m/s (median: 16.4 m/s) [50]. Additionally, the mean value of brachial pulse wave velocity was 1570 ± 281 cm/s in 794 healthy Chinese older adults, and in males 1570 ± 280 cm/s and in females 1580 ± 284 cm/s [51]. Furthermore, sex differences in aging-related arterial stiffness have been demonstrated in previous studies, with a higher pulse wave velocity in females compared to males [51,52]. However, a systematic review and meta-analysis of 167 studies with 509,743 participants reported a higher mean brachial pulse wave velocity in young male adults compared to young female adults, but this trend was reversed for older adults [53]. In the present study, the mean brachial pulse wave velocity was 1602.55 ± 281.65 cm/s, which is within the normal range of the previous studies [50,51], and over 90% of the participants were female. Therefore, the effects of exercise might not have been measurable. Further studies exploring this are warranted.

This study has some limitations that may have affected the findings, which should therefore be interpreted with caution. First, the majority of the participants had a low educational background, and this factor was not controlled for in the study. Educational attainment is associated with a risk of dementia; higher education has been related to a reduced risk of dementia and mild cognitive impairment, with odds ratios of 0.93 and 0.94 per year of school, respectively [54]. Second, the majority of participants were older women (93.48%), and the differences between sexes may be associated with arterial stiffness and cognitive function [55,56]. Third, ambulatory participants were recruited; therefore, the results may not be generalizable to older adults with mild cognitive impairment. Finally, the underlying causes of cognitive decline (i.e., Alzheimer’s disease and vascular cognitive impairment), including potential secondary factors such as vitamin B12 deficiency and hypothyroidism, were not assessed or excluded in this study. Therefore, future studies should specifically focus on primary neurodegenerative processes and the detailed evaluation of mild cognitive impairment causes.

## 5. Conclusions

Moderate-intensity water-based exercise alone or combined with cognitive training has the potential to enhance cognitive performance and cardiovascular fitness, but not reduce arterial stiffness, in older adults. The benefits of water-based exercise interventions include promising cardiovascular fitness and cognitive performance in older adults with mild cognitive impairment. Nevertheless, further studies are required to confirm and extend these findings.

## Figures and Tables

**Table 1:** Sociodemographic and clinical characteristics in the study.

	Total (*n* = 46)	W Group (*n* = 23)	W-COG Group (*n* = 23)	X^2^	*p* Value
Sex					
Male (%)	3 (6.52)	2 (66.67)	1 (33.33)	0.357	1.000
Female (%)	43 (93.48)	21 (43.84)	22 (51.16)		
Educational level				5.248	0.155
Primary (%)	30 (65.22)	16 (53.33)	14 (46.67)		
Secondary (%)	6 (13.04)	1 (16.67)	5 (83.33)		
High school (%)	5 (10.87)	4 (80.00)	1 (20.00)		
Bachelor’s degrees or over (%)	6 (13.04)	2 (33.33)	4 (66.67)		
Underlying disease (%)				2.580	0.461
0 (%)	10 (21.74)	3 (30.00)	7 (70.00)		
1 (%)	18 (39.13)	9 (50.00)	9 (50.00)		
2 (%)	10 (21.74)	5 (50.00)	5 (50.00)		
≥3 (%)	9 (19.57)	6 (66.67)	3 (33.33)		
Exercise				0.521	0.471
<3 days/week (%)	22 (47.83)	12 (54.55)	10 (45.45)		
≥3 days/week (%)	25 (54.35)	11 (44.00)	14 (56.00)		
	Mean ± SD	Mean ± SD	Mean ± SD	*t*(test)	*p* value
Age (years)	68.74 ± 3.52	68.26 ± 3.28	69.43 ± 3.65	−1.147	0.258
BMI (kg/m^2^)	24.25 ± 3.30	24.50 ± 3.91	23.85 ± 2.61	0.661	0.512
MoCA (scores)	22.00 ± 2.19	21.70 ± 2.32	22.29 ± 2.05	−0.933	0.356

SD: Standard deviation, BMI: body mass index; MoCA: Montreal Cognitive Assessment, W group: water-based exercise group, W-COG group: water-based exercise combined with cognitive training group.

**Table 2:** Effect of water-based exercise alone and water-based exercise combined with cognitive training on cardio-respiratory performance and brachial pulse wave velocity over 12-week training.

	W Group (*n* = 23)	W-COG Group (*n* = 23)	Mean Difference ± SE W Group vs. W-COG Group	*p* Value Between Groups
MoCA				
Before mean ± SD	21.70 ± 2.32	22.48 ± 1.89	−0.78 ± 0.62	F (1,44) = 1.576, *p* = 0.216, np^2^ = 0.035
After mean± SD	25.48 ± 3.13	26.57 ± 2.95	−1.09 ± 0.90	F (1,44) = 1.467, *p* = 0.232, np^2^ = 0.032
Mean difference (after-before) ± SE	3.78 ± 0.56	4.09 ± 0.56		
*p* value within group	F (1,44) = 46.142, *p* < 0.001, np^2^ = 0.512	F (1,44) = 53.868, *p* < 0.001, np^2^ = 0.512		
Two MST				
Before mean ± SD	131.87 ± 39.15	135.91 ± 27.43	−4.04 ± 9.97	F (1,44) = 0.167, *p* = 0.687, np^2^ = 0.004
After mean ± SD	154.74 ± 41.10	154.30 ± 28.64	0.44 ± 10.45	F (1,44) = 0.002, *p* = 0.967, np^2^ = 0.000
Mean difference (after-before) ± SE	22.87 ± 5.34	18.39 ± 5.34		
*p*-value within group	F (1,44) = 18.374, *p* < 0.001, np^2^ = 0.295	F (1,44) = 11.884, *p* = 0.001, np^2^ = 0.213		
BaPWV (cm/s)				
Before mean ± SD	1523.91 ± 212.26	1653.00 ± 295.44	−129.09 ± 75.86	F (1,44) = 2.896, *p* = 0.096, np ^2^ = 0.062
After mean ± SD	1531.09 ± 258.19	1695.61 ± 349.23	−164.52 ± 90.56	F (1,44) = 3.300, *p* = 0.076, np^2^ = 0.070
Mean difference (after-before) ± SE	7.17 ± 28.98	42.61 ± 28.98		
*p* value within group	F (1,44) = 0.061, *p* = 0.806, np^2^ = 0.001	F (1,44) = 2.163, *p* = 0.149, np^2^ = 0.047		

SD: Standard deviation, SE: standard error of mean, MoCA: Montreal Cognitive Assessment, Two MST: two-min step test, W group: water-based group, BaPWV: brachial pulse wave velocity, W-COG group: water-based exercise combined with cognitive training group.

## Data Availability

The original contributions presented in this study are included in the article. Further inquiries can be directed to the corresponding author.
